# Novel Radiolabeled Bisphosphonates for PET Diagnosis and Endoradiotherapy of Bone Metastases

**DOI:** 10.3390/ph10020045

**Published:** 2017-05-18

**Authors:** Nina Pfannkuchen, Marian Meckel, Ralf Bergmann, Michael Bachmann, Chandrasekhar Bal, Mike Sathekge, Wolfgang Mohnike, Richard P. Baum, Frank Rösch

**Affiliations:** 1 Institute of Nuclear Chemistry, Johannes Gutenberg University Mainz, Fritz-Strassmann-Weg 2, 55128 Mainz, Germany; pfannkuchen@uni-mainz.de (N.P.); marian.meckel@itg-garching.de (M.M.); 2 Helmholtz-Zentrum Dresden-Rossendorf, Institute of Radiopharmaceutical Cancer Research, Bautzner Landstrasse 400, 01328 Dresden, Germany; r.bergmann@hzdr.de (R.B.); m.bachmann@hzdr.de (M.B.); 3 University Cancer Center (UCC) Carl Gustav Carus, Tumorimmunology, Technical University Dresden, Fetscherstr. 74, 01307 Dresden, Germany; 4 Department of Nuclear Medicine & PET, All India Institute of Medical Sciences, Ansari Nagar, New Delhi 110029, India; csbal@hotmail.com; 5 Department of Nuclear Medicine, University of Pretoria & Steve Biko Academic Hospital, Private Bag X169, Pretoria 0001, South Africa; mike.sathekge@up.ac.za; 6 Diagnostisch Therapeutisches Zentrum, DTZ am Frankfurter Tor, Kadiner Straße 23, 10243 Berlin, Germany; info@berlin-dtz.de; 7 Department of Nuclear Medicine, Center for PET/CT, Zentralklinik Bad Berka, Robert-Koch-Allee 9, 99438 Bad Berka, Germany; richard.baum@zentralklinik.de

**Keywords:** bisphosphonates, bone metastases, diagnosis, therapy, ^68^Ga, ^177^Lu

## Abstract

Bone metastases, often a consequence of breast, prostate, and lung carcinomas, are characterized by an increased bone turnover, which can be visualized by positron emission tomography (PET), as well as single-photon emission computed tomography (SPECT). Bisphosphonate complexes of ^99m^Tc are predominantly used as SPECT tracers. In contrast to SPECT, PET offers a higher spatial resolution and, owing to the ^68^Ge/^68^Ga generator, an analog to the established ^99m^Tc generator exists. Complexation of Ga(III) requires the use of chelators. Therefore, DOTA (1,4,7,10-tetraazacyclododecane-1,4,7,10-tetraacetic acid), NOTA (1,4,7-triazacyclododecane-1,4,7-triacetic acid), and their derivatives, are often used. The combination of these macrocyclic chelators and bisphosphonates is currently studied worldwide. The use of DOTA offers the possibility of a therapeutic application by complexing the β-emitter ^177^Lu. This overview describes the possibility of diagnosing bone metastases using [^68^Ga]Ga-BPAMD (^68^Ga-labeled (4-{[bis-(phosphonomethyl))carbamoyl]methyl}-7,10-bis(carboxymethyl)-1,4,7,10-tetraazacyclododec-1-yl)acetic acid) as well as the successful application of [^177^Lu]Lu-BPAMD for therapy and the development of new diagnostic and therapeutic tools based on this structure. Improvements concerning both the chelator and the bisphosphonate structure are illustrated providing new ^68^Ga- and ^177^Lu-labeled bisphosphonates offering improved pharmacological properties.

## 1. Introduction

Malignant tumors, especially carcinomas of the breast, prostate, and lung, often lead to painful bone metastases. Since complications, like severe bone pain, pathological fractures, spinal cord compression, or hypercalcaemia, distinctly influence the quality of life and, therefore, result in a shorter survival, diagnosis of bone metastases at an early stage, as well as subsequent therapy is of great importance for patients [1,2]. Bone-seeking radiopharmaceuticals are currently used for diagnostic and therapeutic purposes [3].

Bone lesions are characterized by an increase in bone turnover. This increased metabolism of the bone material can be visualized via both single-photon emission computed tomography (SPECT) and positron emission tomography (PET). In contrast to SPECT, PET offers a higher spatial, as well as temporal, resolution [4]. As a SPECT nuclide, ^99m^Tc is predominantly used in the form of ^99m^Tc-labeled bisphosphonate complexes. For PET [^18^F]NaF is used as bone-seeking compound [5,6].

The ^68^Ge/^68^Ga generator system provides a distinguished PET analog of the established ^99m^Tc generator. The daughter nuclide ^68^Ga offers appropriate decay properties (t_1/2_ = 67.7 min, β^+^ = 89%, E_βmax_ = 1.9 MeV) and the generator ensures a long shelf-life with a continuous supply of ^68^Ga [7]. In addition to [^18^F]NaF, ^68^Ga-labeled PSMA radioligands have emerged recently and are currently used for diagnosis of bone metastases as a consequence of prostatic cancer [8]. However, further ^68^Ga-based bone-seeking PET-radiopharmaceuticals have not been established clinically.

The development of radiometal-labeled bisphosphonate-based tracers requires the use of chelators for complexation of trivalent metals. Many research groups across the world are currently undertaking research into complexing bisphosphonate compounds to radionuclides using macrocyclic chelators and aim at identifying a labeled product that has high affinity for bone and offers a high thermodynamic and kinetic stability. For the complexation of Ga(III), DOTA (1,4,7,10-tetraazacyclododecane-1,4,7,10-tetraacetic acid), NOTA (1,4,7-triazacyclododecane-1,4,7-triacetic acid), as well as their derivatives, are commonly used.

For the treatment of disseminated bone metastases, there are two classes of therapeutic bone-seeking radiopharmaceuticals: calcimimetic- and phosphonate-based radiopharmaceuticals. The simplest bone binding radiopharmaceuticals for palliative endoradiotherapy, belonging to the class of calcium mimetics, for example, ^89^Sr, ^32^P, and ^223^Ra. Their localization underlies the same mechanisms as calcium and, therefore, may be unpredictable [9].

**Figure 1:**
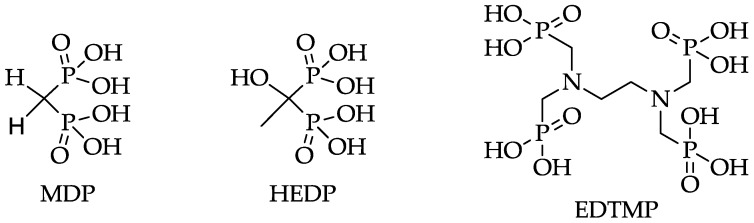
Structures of MDP (methylene diphosphonate), HEDP (1,1-hydroxyethylidene diphosphonate), and EDTMP (ethylenediamine tetra(methylene phosphonic acid)).

Due to the short range of the α-rays emitted by ^223^Ra, an impairment of the red bone marrow can be avoided, while allowing deposition of high-energy doses into the target tissue. The first successful clinical phase III studies showed a low haemotoxicity and prolonged survival in metastatic prostate cancer [10]. However, the consequences of the ^223^Ra decay chain for the body, as well as the influence of the α-rays on the sensitive gastrointestinal tract, remain uncertain [9]. The longer half-lives of nuclides such as ^89^Sr and ^32^P have discouraged their use and have favored nuclides such as ^153^Sm and ^177^Lu with shorter half-lives and lower bone marrow toxicity. The use of ^177^Lu is particularly promising due to its suitable decay properties (t_1/2_ = 6.71 d, β^−^ = 89%, E_βmax_ = 0.5 MeV) and its carrier-free production route [11]. These trivalent nuclides reach regions of increased bone turnover in the form of complexes with phosphonate-containing chelators, like EDTMP (ethylenediamine tetra(methylene phosphonic acid)) (Figure 1). These phosphonate-containing chelators exhibit high thermodynamic stabilities with trivalent nuclides, the acyclic ligands, however, possess lower kinetic stabilities [12]. Nevertheless, radiopharmaceuticals based on phosphonates like EDTMP and HEDP (1,1-hydroxyethylidene diphosphonate) (Figure ) show good results in palliative therapy of painful bone metastases in combination with ^153^Sm, ^177^Lu, ^186^Re, and ^188^Re [13]. ^188^Re has also been shown to have good properties as a therapeutic nuclide due to its appropriate decay characteristics (t_1/2_ = 0.7 d, E_βmax_ = 2.12 MeV) and its generator-based production [14].

However, EDTMP complexes have shown low in vivo stability and an excess of the ligand is routinely applied in order to avoid decomplexation in vivo (>1.5 mg/kg body weight of EDTMP vs. approximately 0.05–0.250 mg BPAMD (4-{[bis-(phosphonomethyl))carbamoyl]methyl}-7,10-bis(car-boxymethyl)-1,4,7,10-tetraazacyclododec-1-yl)acetic acid)) [12,15]. This excess may lead to a blocking of the biological target which could reduce the radiotracer uptake. Furthermore, high amounts of ^152^Sm due to the production route of ^153^Sm can cause a reduction of the dose rate deposited on osseous metastases and, therefore, a lower therapeutic efficiency [16,17]. Using ^177^Lu instead of ^153^Sm increases the specific activity due to the carrier-free production [17], but the low kinetic stability of EDTMP complexes remains problematic.

This review describes the concept of macrocyclic chelate-conjugated bisphosphonates, which are able to circumvent the disadvantages of open-chain chelators, and possible improvements concerning the chosen chelator, as well as the bisphosphonate structure, based on the DOTA bisphosphonate BPAMD.

## 2. Design and Development of Radiolabeled Bisphosphonates

### 2.1. Status Quo

During the last 10 years, the clinical application of bisphosphonates, especially for the treatment of patients with osseous metastases, distinctly increased. Bisphosphonates are analogs of naturally-occurring pyrophosphate. In contrast to pyrophosphate, they are resistant to chemical, as well as enzymatic, hydrolysis due to the substitution of the central oxygen atom by a carbon atom. Their effect is based on two characteristics: they show high affinity for bone material and inhibitory effects on osteoclasts [18]. Binding of bisphosphonates to bone material probably relies on bidentate or tridentate complexation of calcium atoms in the hydroxyapatite depending on the bisphosphonate structure [19].

**Figure 2:**
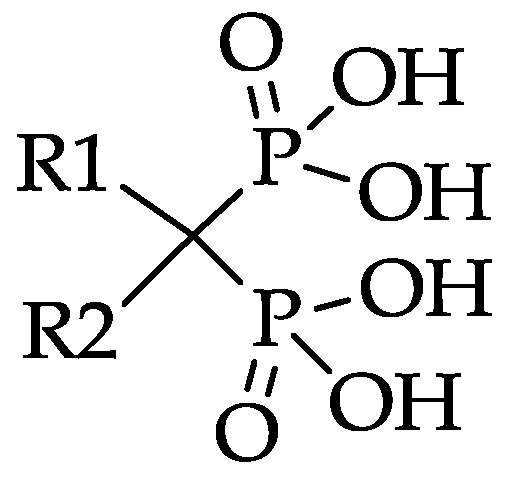
Bisphosphonate structure with variable side chains R1 and R2.

The two side chains on the carbon atom are replaceable and responsible for the activity of the particular bisphosphonate (Figure 2). Substitution of R1 by a hydroxyl or amino group enhances the affinity to hydroxyapatite. Varying the R2 side chain influences the antiresorptive potency [18]. Nitrogen atoms, especially aromatic nitrogen atoms, considerably raise the antiresorptive potency. This is linked to another hydrogen bond between the amine and the hydroxyapatite and the ability to act on biochemical activities, for example, the inhibition of farnesyl pyrophosphate synthase (FPPS) [18].

**Figure 3:**
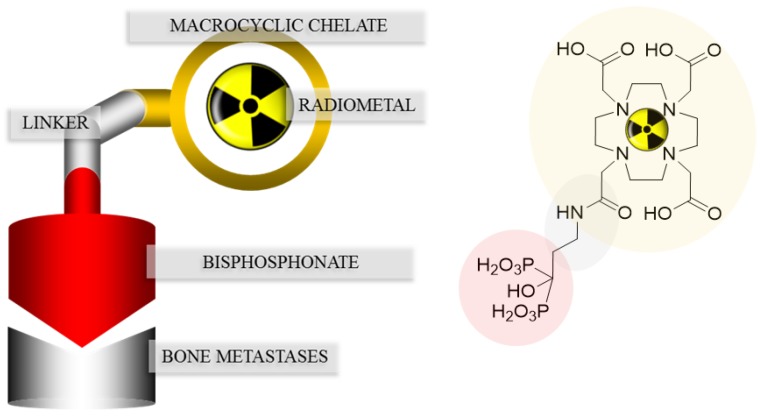
Concept of the combination of a macrocyclic chelator with a bisphosphonate illustrated with DOTA^ZOL^.

In SPECT tracers like [^99m^Tc]Tc-MDP (^99m^Tc-labeled methylene diphosphonate) the phosphonates are responsible for complexation of the radionuclide, as well as binding to the target tissue, which may lead to a decreased uptake in bone metastases [20]. This drawback can be circumvented by complete separation of the chelating unit and the targeting vector, using a macrocyclic chelator for complexation of the radiometal and a coupled bisphosphonate as targeting vector. Figure 3 shows the concept of the combination of a macrocyclic chelator with a bisphosphonate. Depending on the chelator various radionuclides can be complexed, also allowing the combination of diagnosis and therapy in one and the same compound. This theranostic concept already showed excellent results concerning diagnosis and treatment of neuroendocrine tumors using DOTA-TOC (1,4,7,10-tetraazacyclododecan-4,7,10-tricarboxy-methyl-1-yl-acetyl-d-Phe^1^-Tyr^3^-octreotide) radiolabeled with ^68^Ga and ^177^Lu [21].

**Figure 4:**
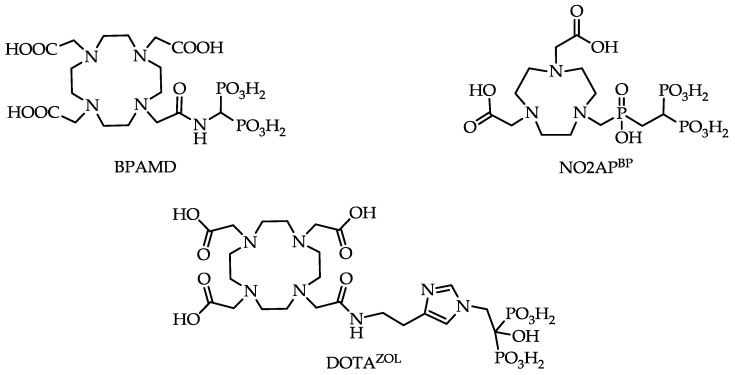
Structures of macrocyclic bisphosphonates BPAMD, NO2AP^BP^ (2,2'-(7-(((2,2-diphosphonoethyl)(hydroxy)phosphoryl)methyl)-1,4,7-triazonane-1,4-diyl)diacetic acid) and DOTA^ZOL^ (2,2',2''-(10-(2-(2-(1-(2-hydroxy-2,2-diphosphonoethyl)-1H-imidazol-4-yl)ethylamino)-2-oxoethyl)-1,4,7,10-tetraazacyclododecane-1,4,7-triyl)triacetic acid).

One of these so-called macrocyclic bisphosphonates is BPAMD (Figure 4), which was initially able to show its high potential in terms of high bone accumulation in ^68^Ga small animal PET experiments [22].

**Figure 5:**
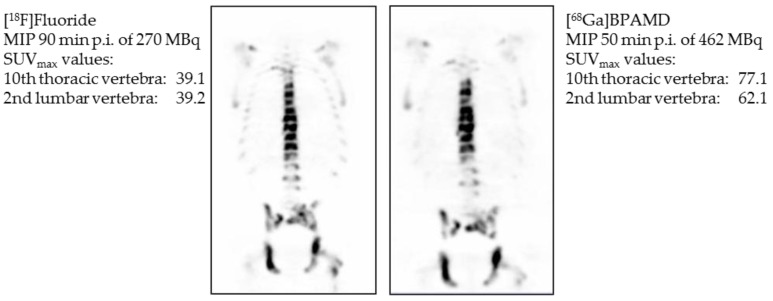
Maximum intensity projection (MIP) 90 min post injection (p.i.) of 270 MBq [^18^F]NaF (**left**) and maximum intensity projection 50 min p.i. of 462 MBq [^68^Ga]Ga-BPAMD (**right**). Comparison of standardized uptake values (SUV) of Th10 and L2 [23].

**Figure 6:**
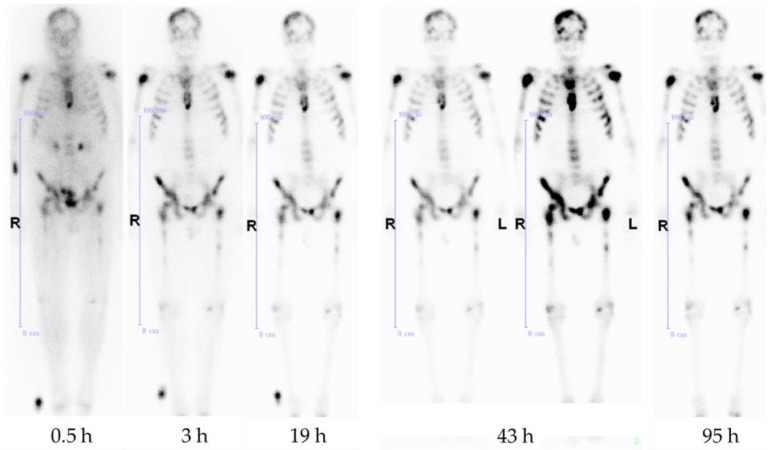
Anterior whole-body scintigraphs of a prostate cancer patient 30 min, 3 h, 19 h, 43 h, and 95 h after application of 3.5 GBq [^177^Lu]Lu-BPAMD, demonstrating abnormal accumulation in the skull, both humeri, ribs, sternum, vertebrae, pelvis, and both femora. The best target-to-background ratio is demonstrated at 43 h p.i.

Later, it also showed good results in the first human applications [23] (Figure 5). The bisphosphonate revealed very high target-to-soft tissue ratios combined with a fast renal clearance. SUVs (standardized uptake values) were comparable with those of the [^18^F]NaF scan, and some metastases even showed higher accumulation of the bisphosphonate. These promising diagnostic examinations finally led to the first therapeutic applications using the β-emitter ^177^Lu instead of ^68^Ga. [^177^Lu]Lu-BPAMD was successfully applied in several patients (Figure 6). It showed a comparable biodistribution as [^68^Ga]Ga-BPAMD, including a good target-to-background ratio and a fast renal clearance. The radiopharmaceutical’s long half-life in the metastases provided high tumor doses which led to a significant reduction in osteoblastic activity of the bone metastases. Furthermore, the therapy did not cause any significant adverse effects [21,24].

A comparative biodistribution study between [^177^Lu]Lu-BPAMD and [^177^Lu]Lu-EDTMP indicated higher bone uptake for [^177^Lu]Lu-BPAMD, as well as a higher target-to-background ratio [25]. This may be attributed to the already-mentioned much higher amount of ligand used for the preparation of [^177^Lu]Lu-EDTMP and the conceivable target blocking.

Despite those promising clinical results, there is still much potential for improvements with regard to radiosynthesis, raising the accumulation in bone metastases and reducing the uptake in healthy tissue. According to Figure 3, both the bisphosphonate and the chelator should be optimized.

### 2.2. Chelator

Chelators based on a polyamino polycarboxylic structure belong to the most efficient ligands and are widely used for the complexation of metal ions. They can be divided into two categories, open chain ligands, such as EDTA (ethylenediaminetetraacetic acid), and DTPA (diethylenetriaminepentaacetic acid) and macrocyclic chelates, such as DOTA or NOTA [26].

DOTA is the most commonly used macrocyclic chelator for PET applications. It is able to complex a variety of isotopes, e.g., ^44/47^Sc, ^111^In, ^177^Lu, ^86/90^Y, and ^225^Ac. It is also used broadly with ^67/68^Ga, which offers the possibility of a theranostic application, as already mentioned using the example of ^68^Ga- and ^177^Lu-labeled DOTA-TOC [27,28]. Nevertheless, DOTA has a comparatively low stability constant for gallium (log K = 21.3) resulting in temperatures of about 95 °C needed for radiolabeling. NOTA, by contrast, exhibits a smaller ring structure and a higher stability constant (log K = 31.0) due to the smaller gallium fitting cavity [28].

**Figure 7:**
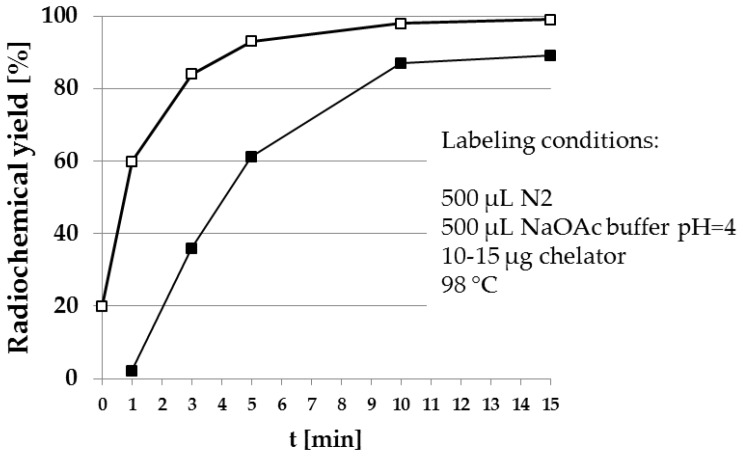
Radiosynthesis of [^68^Ga]Ga-NO2AP^BP^ (□) in comparison to [^68^Ga]Ga-BPAMD (■). The NOTA-based derivative clearly shows higher radiochemical yields within a shorter reaction time.

Figure 7 shows the fast and quantitative ^68^Ga-labeling of the NOTA bisphosphonate NO2AP^BP^ (Figure 4). Comparison with the DOTA-based BPAMD shows the expected faster labeling of NOTA derivatives.

**Figure 8:**
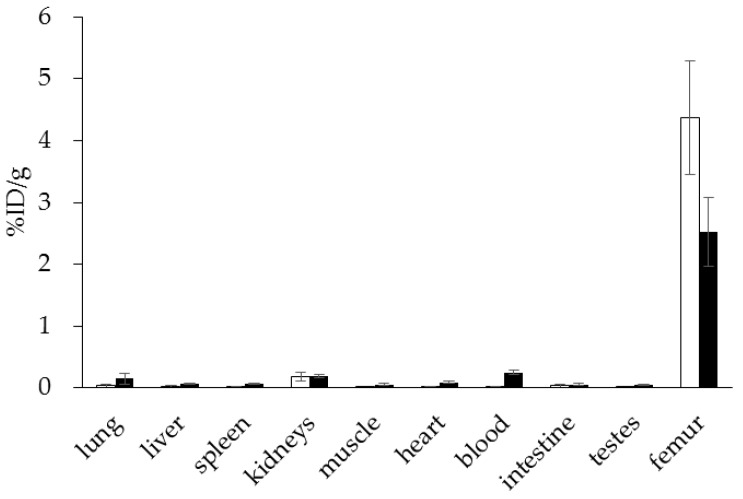
Ex vivo biodistribution of [^68^Ga]Ga-NO2AP^BP^ (□) (3.0 ± 0.1 MBq, *n* = 4) and [^68^Ga]Ga-BPAMD (■) (9.7 ± 1.3 MBq, *n* = 8) in healthy Wistar rats 60 min p.i.

Interestingly, using this NOTA derivative not only provides an improved radiosynthesis, it also exhibits a significantly higher femur accumulation in rats compared with the DOTA derivative BPAMD (Figure 8). This may be explained by differences in charge and physical properties of both complexes. It is a recurrent phenomenon that different chelators provide different in vivo properties with the same target vector [27].

**Figure 9:**
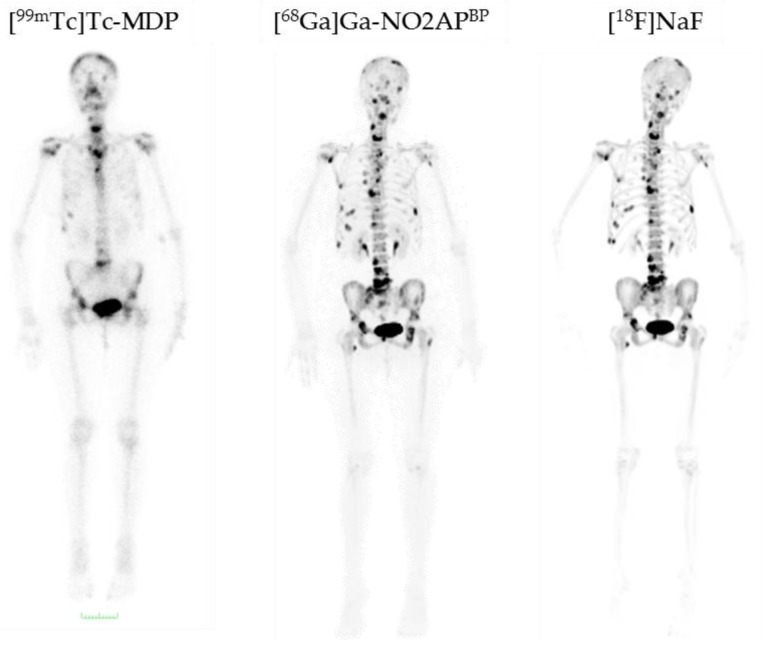
Comparison of [^99m^Tc]Tc-MDP, [^68^Ga]Ga-NO2AP^BP^, and [^18^F]NaF in a breast cancer patient with bone metastases [29]. [^99m^Tc]Tc-MDP demonstrates abnormal uptake only in the skull, cervical vertebra, sternum, two ribs on the right side, and pelvis, while both [^68^Ga]Ga-NO2AP^BP^ and [^18^F]NaF distinctly show more lesions in the skull, cervical, thoracic, lumbar and sacral vertebrae, pelvis, and both femorae. [^68^Ga]Ga-NO2AP^BP^ and [^18^F]NaF are equivalent regarding the number of detected lesions and far superior than [^99m^Tc]Tc-MDP [29].

These positive results were also confirmed in a prospective patient study by Passah et al. comparing [^68^Ga]Ga-NO2AP^BP^, [^18^F]NaF, and [^99m^Tc]Tc-MDP in female breast cancer patients with osseous metastases [29]. Within this study, the NOTA-based bisphosphonate was able to underline its high diagnostic efficiency. Figure 9 shows the PET and SPECT scans of a breast cancer patient. Generally, the PET tracers are able to detect more, as well as smaller, metastases. [^68^Ga]Ga-NO2AP^BP^ revealed a similar detection capability as the gold standard [^18^F]NaF. In selected metastases bisphosphonate uptake was even higher.

In addition to the well-established NOTA derivatives, there is another class of bifunctional chelators appropriate for labeling with ^68^Ga. These so-called DATA chelators are based on 6-amino-1,4-diazepine-triacetic acid and enable more rapid quantitative radiolabeling under milder conditions [30]. The combination of this chelator with next-generation bisphosphonates is also conceivable and might provide a compound of high diagnostic efficiency, as well [31].

### 2.3. Pharmacophoric Group

**Figure 10:**
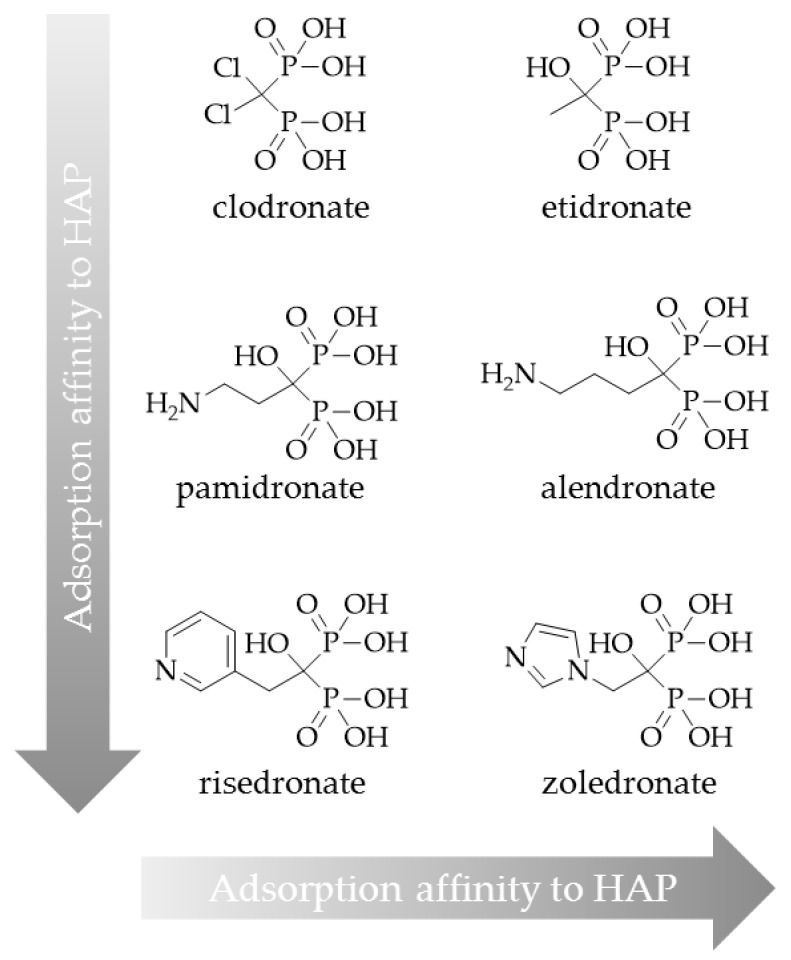
Classification of bisphosphonates according to their adsorption affinity to hydroxyapatite (HAP).

As mentioned above, the side chains on the central carbon atom are responsible for the bisphosphonate’s activity, i.e., in terms of affinity to hydroxyapatite. Using a hydroxybisphosphonate, bearing a hydroxyl group as R1, could lead to higher bone accumulation due to increased affinity for bone material. Furthermore, an aromatic nitrogen atom in the R2 side chain could cause building of another hydrogen bond and thereby also raise bone accumulation (Figure 10) [18].

**Figure 11:**
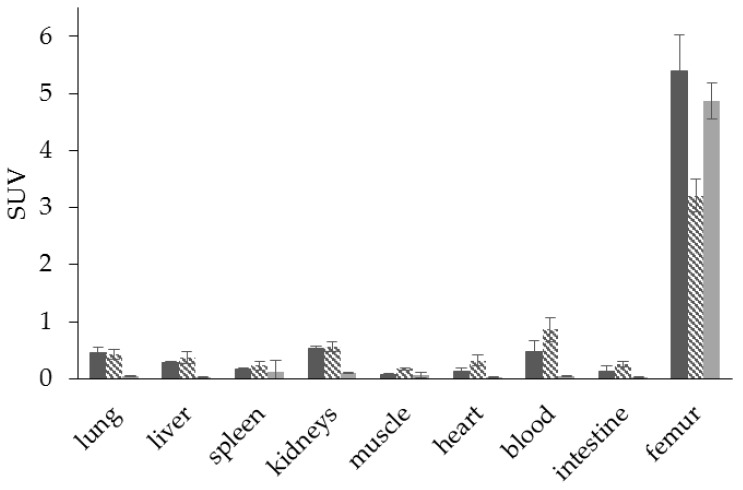
Ex vivo biodistribution of [^68^Ga]Ga-DOTA^ZOL^ (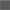) (6.9 ± 0.1 MBq, *n* = 4), [^68^Ga]Ga-BPAPD (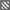) (9.8 ± 0.2 MBq, *n* = 8), and [^18^F]NaF (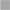) (10.9 ± 0.4, *n* = 4) in healthy Wistar rats 60 min p.i.

**Figure 12:**
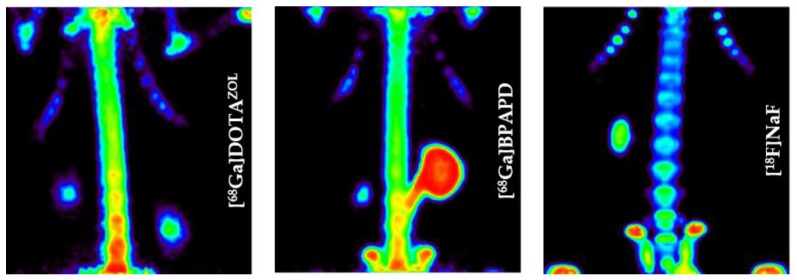
Maximum intensity projection of the thorax region of Wistar rats 60 min p.i. [32].

**Figure 13:**
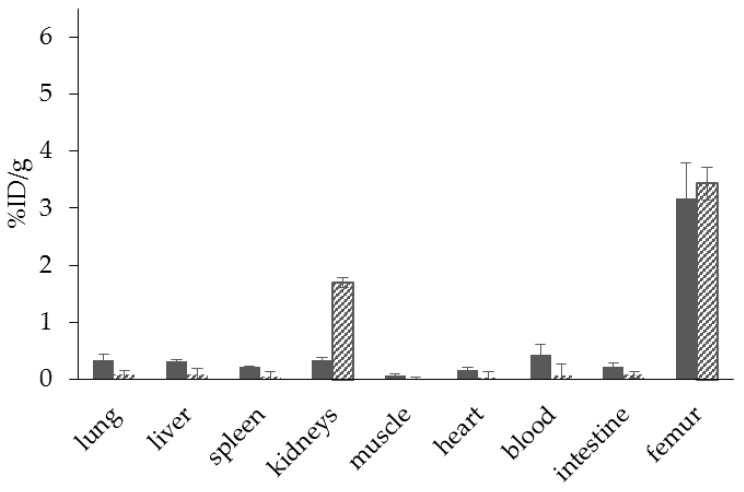
Ex vivo biodistribution of [^68^Ga]Ga-DOTA^ZOL^ (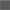) (6.9 ± 0.1 MBq, *n* = 4) and [^177^Lu]Lu-DOTA^ZOL^ (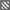) (3.7 ± 0.1 MBq, *n* = 4) in healthy Wistar rats 60 min p.i.

**Figure 14:**
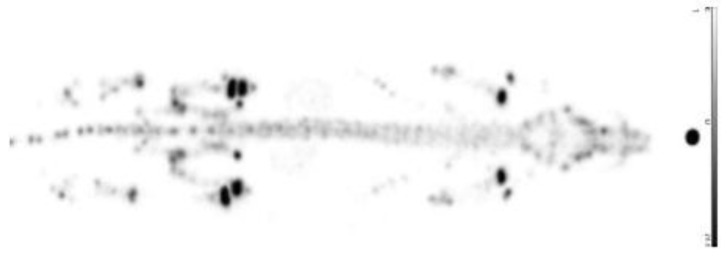
Whole body scintigraphy 60 min p.i. of [^177^Lu]Lu-DOTA^ZOL^ in a healthy Wistar rat [32].

Such a bisphosphonate, like risedronate or zoledronate, would also influence biochemical processes. They possess an inhibiting effect on the FPPS and inhibition of this enzyme causes an increased apoptosis rate [18]. A DOTA-conjugated zoledronate (DOTA^ZOL^, Figure 4) was already labeled with ^68^Ga and ^177^Lu and examined in in vitro and ex vivo biodistribution studies, as well as small animal PET and SPECT studies. [^68^Ga]Ga-DOTA^ZOL^ was compared to [^18^F]NaF and a known DOTA-α-H-bisphosphonate ([^68^Ga]Ga-BPAPD (^68^Ga-labeled (4-{[(bis–phosphonopropyl)carba-moyl]methyl}-7,10-bis-(carboxy-methyl)-1,4,7,10-tetraazacyclododec-1-yl)-acetic acid) (Figure 11 and Figure 12) [32]. [^68^Ga]Ga-DOTA^ZOL^ showed the highest bone accumulation and very low uptake in soft tissue. [^177^Lu]Lu-DOTA^ZOL^ revealed a comparable femur accumulation in ex vivo biodistribution studies in healthy Wistar rats (Figure 13) [32]. Figure 14 shows its high bone accumulation, especially in the high metabolic epiphyseal plates and other joint regions.

Considering the good results of both the ^68^Ga- and the ^177^Lu-labeled derivatives in small animal studies, ^68^Ga- and ^177^Lu-labeled DOTA^ZOL^ seem to offer high potential for theranostic applications. This potential now needs to be proven in clinical studies.

**Figure 15:**
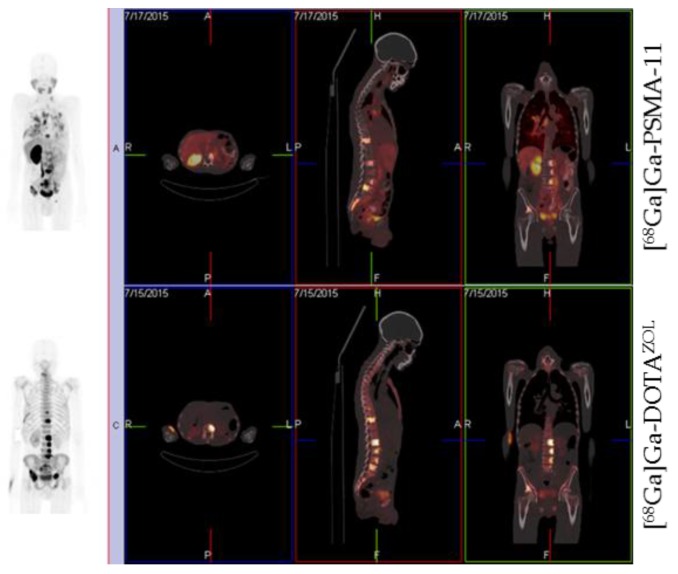
Whole-body PET/CT 60 min p.i. 170 MBq [^68^Ga]Ga-PSMA-11 and 155 MBq [^68^Ga]Ga-DOTA^ZOL^, respectively, in a prostate cancer patient (71 years old, Gleason 4 + 4).

Figure 15 shows a comparison of [^68^Ga]Ga-PSMA-11 and [^68^Ga]Ga-DOTA^ZOL^ in one and the same prostate cancer patient. Both tracers detected multiple skeletal lesions in thoracic and lumbar vertebrae, as well as in the pelvis. Comparison of SUVs revealed an approximately three-fold higher uptake of the bisphosphonate in bone metastases and an approximately three-fold lower uptake in normal tissue organs, exemplifying the bisphosphonate’s better target-to-background ratio.

**Figure 16:**
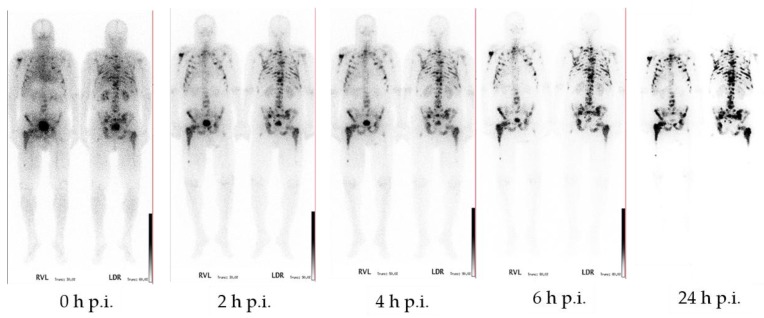
Anterior and posterior whole-body scintigraphs of [^177^Lu]Lu-DOTA^ZOL^ in a prostate cancer patient. 0 min, 2 h, 4 h, 6 h, and 24 h after application of 1 GBq [^177^Lu]Lu-DOTA^ZOL^ demonstrating multiple intense accumulation in the axial skeleton and both humerae and femorae. The target-to-background ratio is progressively better in later images and best at 24 h p.i.

[^177^Lu]Lu-DOTA^ZOL^ has been used in ten patients with bone metastases for dosimetry studies (data to be published). Whole body scintigraphic images were acquired at different time points after injection. Within this study it showed a fast renal clearance and a high target-to-background ratio, and was able to confirm the results of the ex vivo biodistribution studies (Figure 16).

## 3. Conclusions

The combination of novel bisphosphonates with macrocyclic chelators provides promising tracers for diagnosis, therapy, and also theranostics of bone metastases.

Currently, the most potent ^68^Ga-bisphosphonate is [^68^Ga]Ga-NO2AP^BP^, which enables quantitative radiolabeling and exhibits very high accumulation in bone metastases 30–60 min after injection, as well as a fast blood clearance and very low uptake in soft tissue. It is superior to [^99m^Tc]Tc-MDP and comparable to [^18^F]NaF. 

DOTA bisphosphonates are eminently suitable for labeling with ^177^Lu. [^177^Lu]Lu-BPAMD has proved valuable in clinical application. The low-energy β^−^ emission hardly reaches the bone marrow and only a low or no haematotoxicity was observed. The good target-to-background ratio, that all examined bisphosphonates have in common, is also advantageous for therapeutic applications due to reduced radiation dose for non-target tissue. 

Due to further developments regarding the chemical structure of these macrocyclic bisphosphonates, new ^68^Ga- and ^177^Lu-labeled bisphosphonates possessing improved pharmacological properties are expected. Zoledronate based bisphosphonates appear to be the most potent radiotracers with regard to bone lesions. Thus, DOTA^ZOL^ for example may be a potent conjugate for theranostics of bone metastases.
